# Microbe–Anode Interactions: Comparing the impact of genetic and material engineering approaches to improve the performance of microbial electrochemical systems (MES)

**DOI:** 10.1111/1751-7915.14236

**Published:** 2023-02-18

**Authors:** Edina M. Klein, Melanie T. Knoll, Johannes Gescher

**Affiliations:** ^1^ Institute of Technical Microbiology University of Technology Hamburg Hamburg Germany

## Abstract

Microbial electrochemical systems (MESs) are a highly versatile platform technology with a particular focus on power or energy production. Often, they are used in combination with substrate conversion (e.g., wastewater treatment) and production of value‐added compounds via electrode‐assisted fermentation. This rapidly evolving field has seen great improvements both technically and biologically, but this interdisciplinarity sometimes hampers overseeing strategies to increase process efficiency. In this review, we first briefly summarize the terminology of the technology and outline the biological background that is essential for understanding and thus improving MES technology. Thereafter, recent research on improvements at the biofilm–electrode interface will be summarized and discussed, distinguishing between biotic and abiotic approaches. The two approaches are then compared, and resulting future directions are discussed. This mini‐review therefore provides basic knowledge of MES technology and the underlying microbiology in general and reviews recent improvements at the bacteria–electrode interface.

## Graphical abstract

Microbial electrochemical systems (MESs) are a highly versatile platform technology with a particular focus on power or energy production. This rapidly evolving field has seen great improvements both technically and biologically, but this interdisciplinarity sometimes hampers overseeing strategies to increase process efficiency. In this review, we summarize and discuss research on improvements at the biofilm‐electrode interface distinguishing between biotic and abiotic approaches.

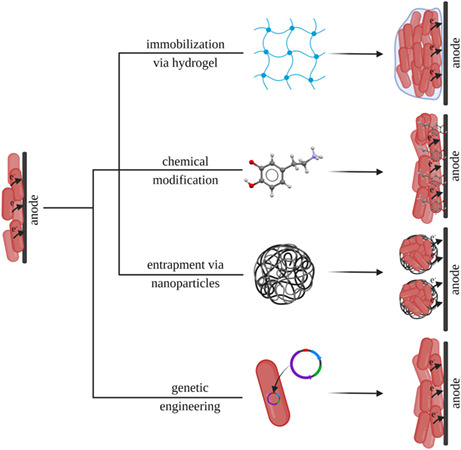

## INTRODUCTION

Bioelectrochemical systems (BESs) convert chemical into electrical energy or vice versa. When microbial organisms are used as catalysts in these systems, they are referred to as microbial electrochemical systems (MESs). According to the direction of the electron flux, MESs are mostly divided into anodic and cathodic systems. MESs can be applied to use a reductive current to power a catalytic process on the cathode side. Here, for instance, autotrophic microorganisms can be utilized for production processes based on carbon dioxide as substrate. In contrary, MESs can also be used to harvest a positive current of electrons flowing to the anode and in this mini‐review, we focus solely on the interaction between biofilms and anode surfaces, and therefore only anodic BESs are covered in more detail in the subsequent sections. As the rapidly developing field of reactor development is also not in the focus of this review, we would like to refer at this point to other references (e.g., Mohanakrishna, Kalathil, & Pant, 2018; Kadier et al., 2016; Bhargavi et al., 2018).

### Anodic bioelectrochemical systems: principals, variants and applications

**FIGURE 1:**
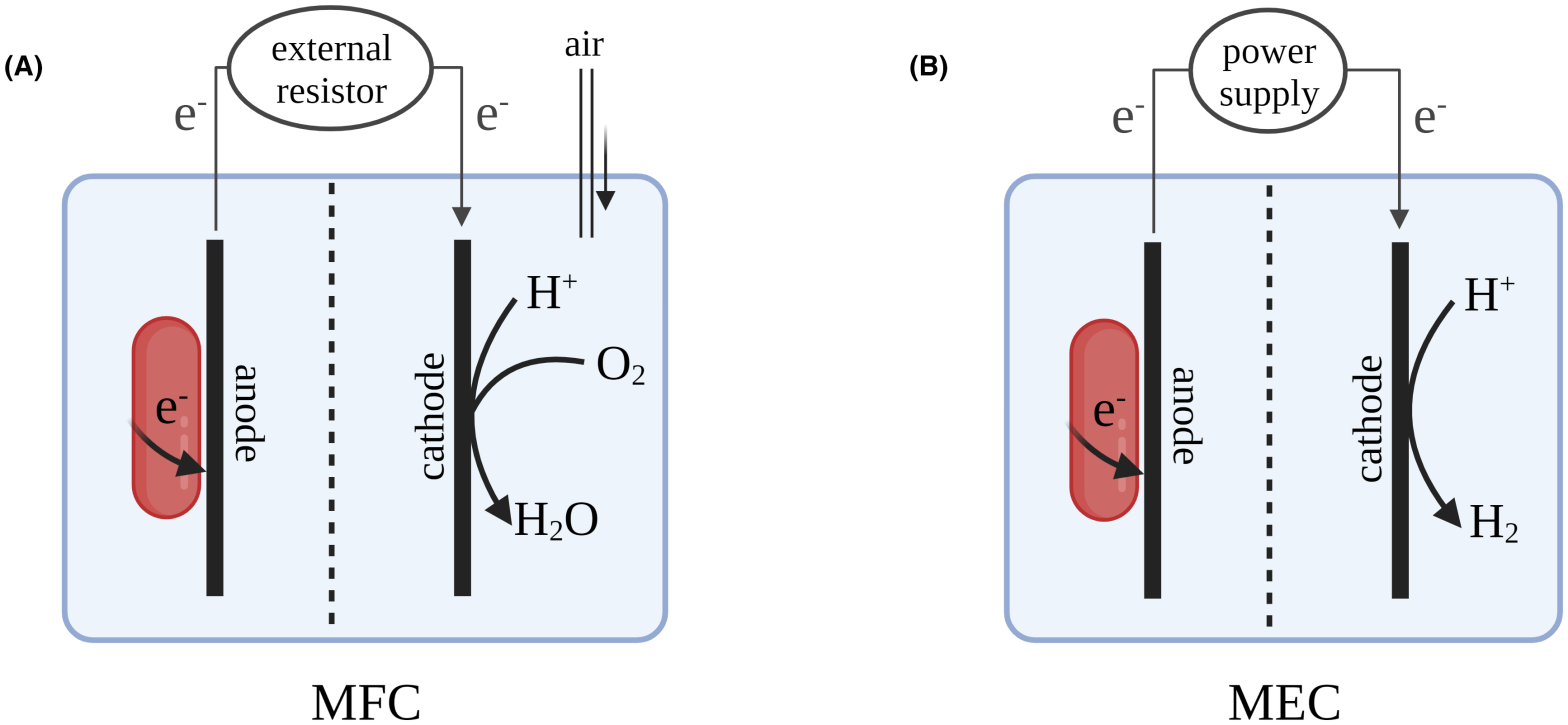
Schematic illustration of the microbial fuel cell (MFC, A) and the microbial electrolysis cell (MEC, B). Microbes usually grow in the form of a biofilm on the anode and transfer their electrons to the electrode. While in MFCs an external resistor is part of the electrical circuit between the bioanode and cathode, in MECs additional power input allows for the production of hydrogen (H_2_).

In anodic MESs, microorganisms oxidize organic and/or inorganic substrates (electron donor) and transfer the generated electrons to the anode (electron acceptor), whereas the protons are released to the electrolyte. The electrons flow through a circuit either through an external electrical resistance (microbial fuel cell, MFC; see Figure 1A) or an external power supply system (microbial electrolysis cell, MEC; see Figure 1B) to the cathode. In an MFC, the voltage resulting from the oxidation of the substrate at the anode and the cathodic reaction is used to generate an electric current, and no external energy or power input is provided additionally. In most cases, oxygen (O_2_) is reduced at the cathode, but other electron acceptors such as ferricyanide or persulfate can be used as well (Ucar, Zhang, & Angelidaki, 2017). In contrast, MECs are supplied with an additional potential, which is sufficient to reduce protons to hydrogen gas at the cathode. Hence, the current production in MECs directly correlates with hydrogen gas production at the cathode (Kadier et al., 2019).

The range of applications for anodic MESs is surprisingly wide. An especially well‐known application of anodic BESs is their (intended) use in wastewater treatment plants for organic carbon elimination. Wastewater is provided as organic substrate for the microorganisms. Thus, wastewater treatment, energy generation and, in some cases, resource recovery can be combined (Koul et al., 2022). In fact, BES technology was already successfully applied to various types of wastewater (Ding, Cheng, Yu, & Huang, 2017; Hiegemann et al., 2019; Lu et al., 2017; Martinucci et al., 2015; Trapero, Horcajada, Linares, & Lobato, 2017), but the ideal performance has not been attained yet due to certain shortcomings. These include, among others, membrane and cathode fouling leading to short lifespan (Daud et al., 2019; Kiely, Rader, Regan, & Logan, 2011), low production rates (Clauwaert et al., 2008), high costs, and limited efficiencies (Do et al., 2018; He et al., 2017).

Another promising variation of anodic MESs are microbial solar cells (MSCs). MSC is a collective name for systems that integrate photosynthetic organisms to harvest solar energy and convert this energy either directly or by interaction with a secondary electroactive organism into electricity and chemical compounds (Apollon et al., 2021; Hamelers et al., 2010; Strik et al., 2011).

Further applications of anodic BESs include the use for (I) microbial electrolysis for carbon capture (MECC; Lu, Huang, Rau, & Ren, 2015), (II) anode‐assisted fermentation of value‐added substances (Beblawy, Philipp, & Gescher, 2020; Härrer, Elreedy, Ali, Hille‐Reichel, & Gescher, 2023; Palma‐Delgado, Paquete, Sturm, & Gescher, 2019), and (III) biosensors (Golitsch, Bücking, & Gescher, 2013; Ivars‐Barceló et al., 2018; Modin, Wang, Wu, Rauch, & Fedje, 2012; Pasternak, Greenman, & Ieropoulos, 2017) among others.

### Electroactive microorganisms (EAMs) as catalyst in microbial electrochemical systems (MESs)

Electroactive microorganisms (EAMs) are the catalysts of MESs, and fundamental knowledge about their metabolism is consequently crucial for the improvement of MES performance. All established model organisms and most of the organisms reported to interact with electrodes overall belong to the bacteria. However, it is worth mentioning that there have been also reports on electroactive archaea and even eukaryotes (Pillot et al., 2018; Raghavulu, Goud, Sarma, & Mohan, 2011; Yilmazel, Zhu, Kim, Holmes, & Logan, 2018). Nevertheless, we will focus here on bacterial systems as in‐depth biochemical knowledge on extracellular electron transfer (EET) pathways operating in organisms belonging to other kingdoms is sparse. Anodic respiring organisms conduct EET as part of their natural anaerobic metabolism (Logan, 2009; Lovley, 2006). The microbial electron transfer chains transfer electrons from low‐potential electron donors to acceptors with a more positive redox potential through a series of redox reactions. So far, two EET strategies were elucidated in more detail: direct electron transfer (DET) by surface‐bound cytochromes or microbial nanowires and mediated electron transfer (MET) by electron shuttling molecules (Beegle & Borole, 2018; Haddock & Schairer, 1973; Logan et al., 2006; Reguera, 2018). As these mechanisms are summarized in several recent reviews, we do not examine them in more detail here (Beegle & Borole, 2018; Light et al., 2018; Lovley, 2017a, 2017b; Reguera, 2018).

In most MESs, a minority of the microorganisms thrives planktonically in the electrolyte, while most of the community grows directly on the electrode as an electroactive biofilm. The cells are embedded in a self‐produced matrix of extracellular polymeric substances (EPSs) and adhere to each other and/or the electrode surface (Vert et al., 2012). Applying electroactive biofilms as catalysts in MESs has a wide variety of benefits, such as the high resistance of biofilms to harsh and/or changing environmental conditions. Harsh conditions for a microbial cell may include high flow rates (shear forces), toxic‐ or growth‐inhibiting substances, high or varying substrate concentration, and especially rapid changes of process conditions in general (Brunner, Klessing, Dötsch, Sturm‐Richter, & Gescher, 2019; Flemming et al., 2016; Härrer et al., 2023; Yin, Wang, Liu, & He, 2019). Biofilms are composed of a large number of cells with densities ranging from 10^8^ to 10^11^ cells g^−1^ wet weight (Balzer, Witt, Flemming, & Wingender, 2010; Morgan‐Sagastume, Larsen, Nielsen, & Nielsen, 2008). This immobilization of many cells within a small volume is a significant advantage, especially for a biotechnological application on an industrial scale. Additionally, biofilms allow for dynamic nutrient exchange and simplify cellular communication (Costerton, Lewandowski, Caldwell, Korber, & Lappin‐Scott, 1995; Donlan, 2002). Stable gradients of substrates and products can form within the biofilm matrix, which can lead to a differentiation and adaptation of the microorganisms' physiology to the specific conditions within the biofilm. If mixed cultures are cultivated, these gradients will provide a variety of habitats for a diversity of different organisms that thrive in an interactive manner (Flemming et al., 2016). Over time, however, as the biofilm grows in height, several factors need to be considered. These include in particular that diffusion limitations may occur (Renslow et al., 2010) and that conductivity decreases with increasing thickness. As biofilm thickness increases, DET through outer membrane cytochromes becomes less relevant due to the limited number of cells that can come in direct contact with the anode. Also MET via electron shuttles at commonly observed concentrations limits the rate of EET and the thickness of the biofilm (Torres et al., 2010). However, electron transfer mediated by conductive pili or nanowires allows long‐range electron transfer through multiple cell layers and thus the growth of thick biofilms in which many cell layers actively contribute to current production (Malvankar et al., 2012; Reguera et al., 2006; Torres et al., 2010). In this context, Malvankar et al. (2012) postulated a correlation between conductivity based on nanowires of *G. sulfurreducens* biofilms and current density, hypothesizing that increasing conductivity of the biofilm itself could be more relevant for performance improvement than increasing biofilm thickness in general. Interestingly, in terms of maximum biofilm thickness, research here suggests different limits for EAM biofilms. For example, Dhar et al. (2017) achieved a maximum biofilm thickness for *G. sulfurreducens* of 80 μm, while Renslow et al. (2013) could cultivate a 400‐μm‐thick biofilm using the same organism. The first study focused on the influence of buffer capacitance of a biofilm and showed that conductivity and current production decrease significantly at lower buffer concentrations. Furthermore, Renslow et al. studied the penetration depth of acetate as electron donor and demonstrated that this depth is limited to 100 μm, suggesting that in a 400‐μm biofilm the majority of cells are metabolically inactive due to acetate depletion and act as electrical conduit for the active top layer. In conclusion, both studies postulate that the productivity of *G. sulfurreducens* biofilms could be negatively influenced at a thickness above 80 to 100 μm. For biofilms formed from mixed cultures, a similar value of around 100 μm biofilm thickness has been postulated by Virdis, Millo, Donose, and Batstone (2014). The authors investigated the reduction state of *c*‐type cytochromes and revealed that the cytochromes were homogeneously oxidized up to a thickness of 100 μm, while above 100 μm an accumulation of electrons was observed within the biofilm.

**FIGURE 2:**
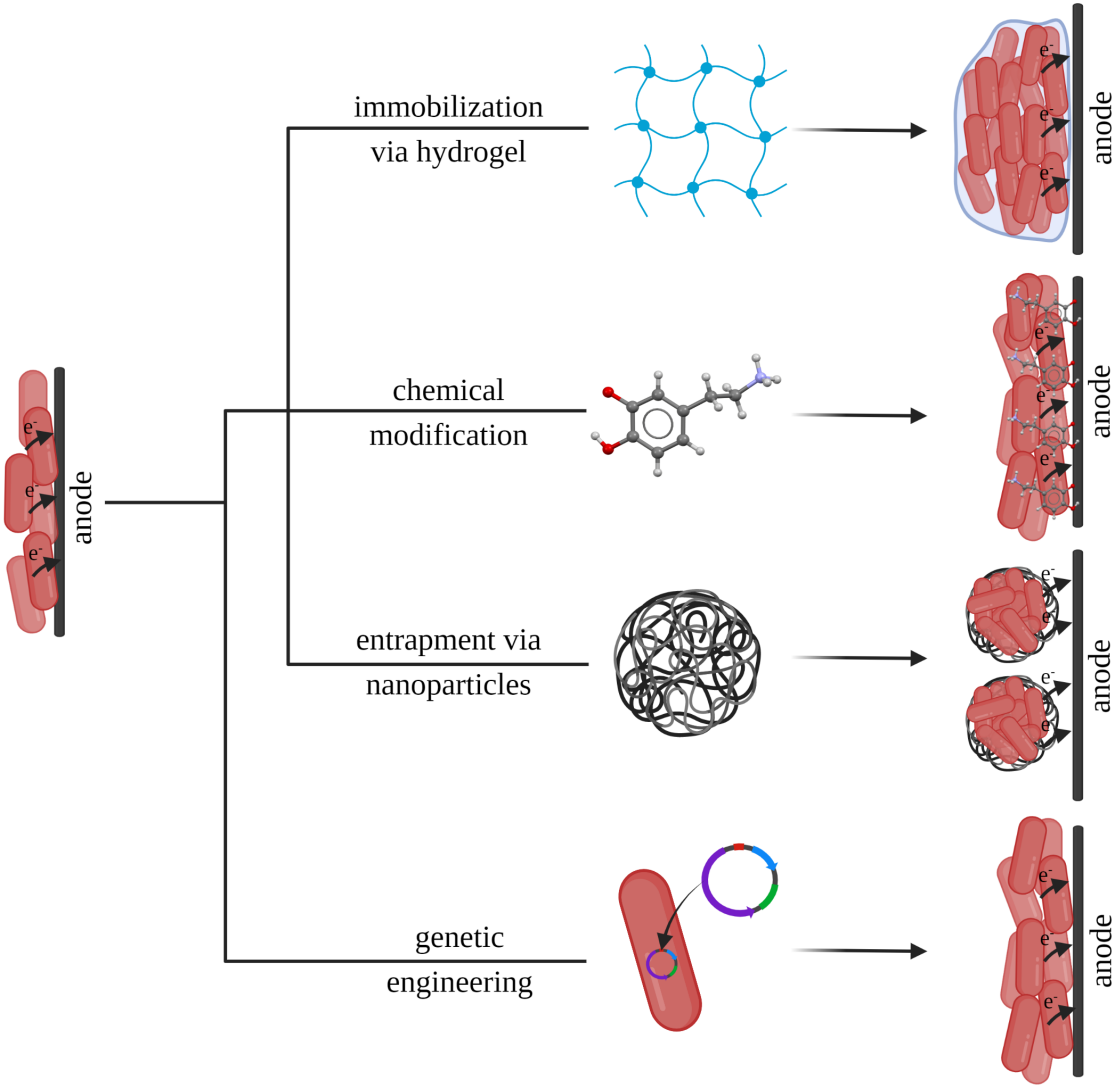
Optimization strategies to improve the performance of bioelectrochemical systems. The interaction between biofilm and anode is altered by genetic engineering or modifications using nanoparticles, chemical compounds, and hydrogels. These improvements include better biofilm formation, higher conductivity throughout the biofilm, better adhesion processes due to more attachment sites, larger electrode surface area or lower electrical resistance, and improved biocompatibility between electrode material and bacteria.

In conclusion, the interaction of cells within electroactive biofilms as well as the interface between microorganisms and electrode are of outstanding importance for improving BES performance. Therefore, it is not surprising that research is currently aimed at improving this interface. The optimization of biofilm–electrode interaction can essentially be divided into four areas: (I) genetic modification, (II) immobilization of organisms using hydrogels, and biofilm as well as electrode engineering using (III) nanoparticles, or (IV) chemical compounds (Figure 2). This review summarizes recent advances in these optimization approaches and provides a tabular overview of the improvement factors in these categories.

## OPTIMIZATION OF ELECTROACTIVE ANODIC BIOFILMS AT THE GENE LEVEL

Targeting the microorganism/biofilm–electrode interface to improve current production or energy yields in MES is an often‐addressed approach. An investigation on the level of the impact of individual genes is hereby a very systematic concept, which contributes to a gain of fundamental understanding of EET and additionally to the improvement of the MES technology itself. Considering recent publications reporting improved current production or power densities through genetic engineering, or at least reporting one or more genes associated with an improvement, it is striking to find research items almost entirely only involving different *Shewanella* and *Geobacter* species (see Table 1). These genera are the best understood organisms concerning EET; hence, a majority of research groups focuses on these model organisms.

**TABLE 1:** Comparison of approaches for improving biofilm–electrode interaction at the gene level. Improvement factor (IF) in regard to a = power density (voltage × resistance^−1^ / power × area^−1^), b = specific power density (power density × optical density), c = maximum current density (current × area^−1^), d = maximum power density, e = final current density, f = mean current density (integrated current × time^−1^ × area^−1^), g = maximum current and h = steady‐state current density. In each case, the corresponding absolute value is given in parentheses.

Organism	Improvement strategy	Anode	IF and further improvements	Reference
*Shewanella oneidensis* MR‐1	Overexpression of *cymA* (pBBR1MCS‐2/ *cymA*)	Plain porous carbon paper	1.×^a^ (0.13 mW) 1.17×^b^ (0.18 W m^−2^ OD^‐^^1^)	Vellingiri et al. (2019)
	Replacement of the genes *nrfA*, *ccpA*, *napB* and *napA* by *cctA* (*nrfA::cctA*, *ccpA::cctA*, *napB::cctA*, *napA::cctA*)	Graphite felt	1.2×^c^ 1.47^f^ 1.7× ferric iron reduction rate (0.95 mM h^−1^)	Palma‐Delgado et al. (2019)
	Deletion of *napB*, *fccA* and *tsdB*, and overexpression of *cctA* (Δ*napB* Δ*fccA* Δ*tsdB* pHGEN‐Ptac‐*cctA*)	Carbon cloth	3.62×^d^ (0.44 W m^−2^)	Sun et al. (2021)
	Introduction of a plasmid with entire flavin biosynthesis gene cluster (*ribADEHC*) cloned from *Bacillus subtilis* to already established non‐competent biofilms in situ by ultrasound‐mediated DNA delivery (UDD) technique (PYYDT‐C_5_‐*ribADEHC*)	Carbon cloth	Classic transformation: 2.3×^h^ (0.32 ± 0.003 A m^−2^) 2.0×^d^ (0.053 ± 0.012 W m^−2^) UDD technique: 1.6×^e^ (0.22 ± 0.012 A m^−2^) 1.46× flavin concentration (103.3 ± 8.3 μM)	Ng et al. (2021)
*Shewanella oneidensis* MR‐1	Deletion of the prophage λ	Graphite felt	1.34×^f^ (0.067 A m^−2^) 2.3× cell density on the anode surface (1.1E+11 cells)	Bursac et al. (2017)
	*speC* overexpression (pBAD‐*speC*)	Graphite felt	1.89×^f^ (0.208 A m^−2^)	Edel et al. (2021)
	Heterologous expression of flavin biosynthesis pathway from *Bacillus subtilis*	Carbon cloth	14.21×^d^ (0.23 W m^−2^)	Yang et al. (2015)
*Shewanella decolorationis*	Δ*sorA*	Graphite plate	1.25×^g^ (0.2 A m^−2^)	Kong et al. (2020)
*Shewanella carassii*‐D5	Overexpression of the *ribADEHC* cluster from *Bacillus subtilis* (pYYDT‐C_5_‐*rib*‐*ADEHC*)	Carbon cloth	1.6×^d^ (0.18 W m^−2^)	Yang et al. (2022)
*Geobacter sulfurreducens*	Deletion of genes encoding a PilZ domain: Ten genes predicted Five genes well conserved among the completed genomes of five *Geobacter* species Attempts to delete these five genes only yielded two viable strains (ΔGSU1240 and ΔGSU3263) ΔGSU1240 was chosen for further studies because the strain readily produced aggregated when grown with fumarate as the electron acceptor	Graphite rod	1.7×^a^ (1.3 W m^‑2^) 1.5×^c^ (2.5 A m^‑2^) 6× biofilm conductivity	Leang et al. (2013)
*Geobacter sulfurreducens*	Isolation of 9 *Geobacter sulfurreducens* from biofilms formed on an anode in a BES in which river sediment was used as an inoculum, of which especially YM18 shows an increased maximum current density in comparison with the type strain PCA YM18 compared to PCA: *omcB*, *xapD*, *spc*, *ompJ* not present *spc*::YM18_1458 YM18 apparently has intact gene clusters for LPS and CPS Two regions encoding CRISPR/Cas systems were missing in YM18	Graphite stick anode	In comparison with type strain PCA: YM18: 1.62×^c^ (9.29 A m^−2^)	Fujikawa, Ogura, Hayashi, and Inoue (2021), Fujikawa, Ogura, Ishigami, et al. (2021)
	Overexpression of nanowire genes *pilA*, *omcZ*, *omcS*, *omcT* (pOEpilA, pOEomcZ, pOEomcS and pOEomcS). All result in a similar increase in current/power density in comparison with the control strain.		1.32–1.62×^c^ (2.4–2.9 A m^−2^) 2.62–2.98×^d^ (1.4–1.6 W m^−2^)	Wang et al. (2022)
*Shewanella oneidensis* & *Klebsiella pneumoniae*	Consortium of both organisms that was genetically optimized for a broader substrate spectrum. Glycerol as feed substrate was degraded to lactate by *K. pneumoniae*. Genetic engineering of *K. pneumoniae* included deletion of the native alcohol dehydrogenase and heterologous expression of a lactate dehydrogenase and a lactate transporter from *Lactobacillus bulgaricus* and *E. coli*, respectively.	Carbon cloth	2.44×^d^ (0.02 W m^−2^)	Li et al. (2018)

### Genetic optimizations for *Shewanella* species

**FIGURE 3:**
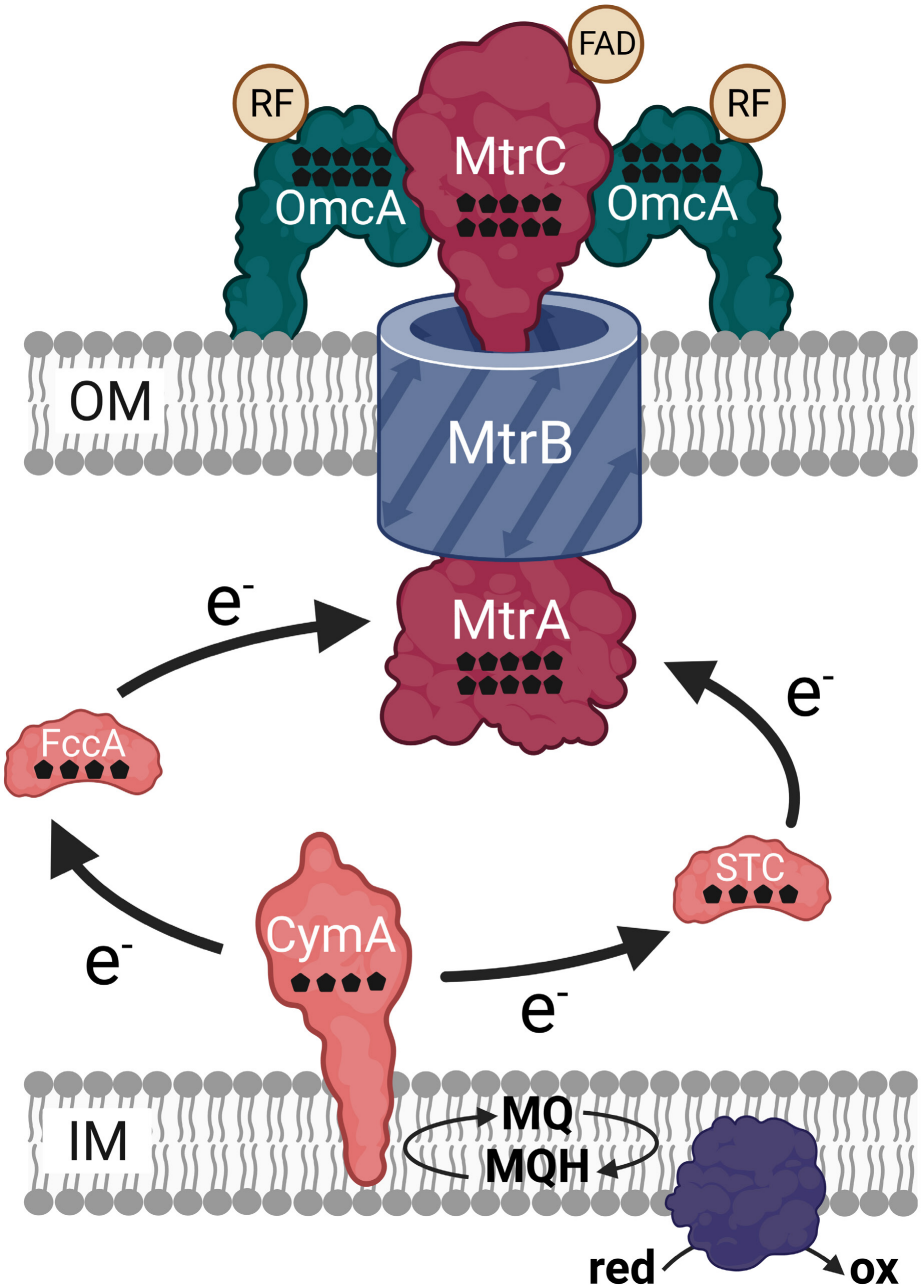
Scheme of the protein network for extracellular electron transfer (EET) in *S. oneidensis*. Black pentagons indicate the number of heme cofactors in *c*‐type cytochromes, IM stands for inner membrane, OM stands for outer membrane, the purple protein in the inner membrane represents an oxidoreductase, and FAD and RF stand for flavin adenine dinucleotide and riboflavin, respectively.

Since several strains belonging to the genus *Shewanella* are almost as genetically accessible as *Escherichia coli*, it is not surprising that most groups focus on *Shewanella* when it comes to genetic engineering. The current knowledge on the biochemistry of EET is mostly based on experiments with *S. oneidensis* and is summarized in Figure 3. Respiratory electrons are transferred via the cytoplasmic membrane‐bound *c*‐type cytochrome CymA into the periplasm (Gralnick & Newman, 2007). Electron transfer via either the tetraheme cytochrome FccA or STC connects the electron transfer chain to the outer membrane (Alves et al., 2015). Here, a heterotrimeric complex of two decaheme cytochromes and a β‐barrel protein forms a conductive pore through the outer membrane (Hartshorne et al., 2009; Ross et al., 2007). Terminal electron transfer via MtrC is supported by the cofactor riboflavin that can also be used as electron shuttle, when released by the cell (Okamoto, Nakamura, Nealson, & Hashimoto, 2014; Von Canstein, Ogawa, Shimizu, & Lloyd, 2008). In 2019, Vellingiri et al. achieved a 1.18‐fold increase in maximum power of *Shewanella oneidensis* in MFCs by overexpressing the *c*‐type cytochrome *cymA*. The overexpression also resulted in a higher growth rate in an MFC compared to the progenitor strain. A higher electrochemical activity could be demonstrated by cyclic voltammetry and linear sweep voltammetry, indicating that more respiratory electrons were transferred to the electrode. CymA transfers electrons from the inner membrane to various terminal electron acceptors and is essential for the anaerobic respiration of *Shewanella* sp. (Myers & Myers, 1997). According to current knowledge, respiratory electrons are transferred to the menaquinone pool in the cytoplasmic membrane. From there, CymA transports the electrons to a network of periplasmic redox proteins, which is dominated by *c*‐type cytochromes (Alves et al., 2015; Sturm et al., 2015). Interestingly, most of the anaerobic electron transfer chains are CymA‐dependent, and therefore the results of Vellingiri et al. (2019) fit well in this context.

The hypothesis that reducing the complexity of the electron transfer network in the periplasm of *S. oneidensis* while overexpressing a periplasmic electron shuttle could lead to an increase in the electron transfer rate was postulated by Palma‐Delgado et al. (2019). Motivated by this, Palma‐Delgado et al. (2019) replaced four genes (namely *nrfA*, *ccpA*, *napA* and *napB*) by the periplasmic *c*‐type cytochrome *cctA* (STC). This led to a 1.23‐fold increase in maximum current density in a MEC system and a 1.7‐fold increased ferric iron reduction rate. A rather comparable study was conducted by Sun, Lin, Yu, Cheng, and Gao (2021) in an MFC instead of a MEC system. The genes *napB*, *fccA* and *tsdB* were deleted, and *cctA* was overexpressed from a plasmid resulting in a 3.62‐fold increase in maximum power density.

The studies discussed so far primarily address periplasmic heme‐containing proteins. A study, which in contrast to this investigates a periplasmic non‐heme protein, reports on a 1.25‐fold increase in maximum power density by deleting *sorA* (Kong et al., 2020). Since biofilm formation was also increased, this leads to the assumption that biofilm volume and increased power output are related. *sorA* encodes a sulfite dehydrogenase molybdenum‐binding subunit in *Shewanella decolorationis*. Interestingly, this effect seems to be electrode‐specific, since no effect was seen on respiration with oxygen, fumarate, azo dye, or ferric citrate. The study gives evidence for an inhibitory role of SorA on EET to an electrode but does not really speculate on the underlying reason for this.

Interestingly, Bursac, Gralnick, and Gescher (2017) achieved a 1.34‐fold increase in mean current density without applying any alterations to the EET itself. The authors aimed to construct a more stable chassis strain for microbe–electrode interaction, and therefore deleted one, two, or all three prophages in the genome of *S. oneidensis*. The λ‐prophage deletion mutant showed a 1.34‐fold increase in mean current density in a MEC system compared to the wild type and furthermore showed a 2.3‐fold increase in cell density on the anode surface. It was stated that this improvement was due to less prophage‐induced cell lysis. The strain was further engineered to produce acetoin from lactate, in an MEC system. By using electrode‐assisted fermentation, it was possible to overcome the obstacle that the average oxidation state of substrate and products must be identical in the absence of an external electron acceptor.

Since the assumption has already been made above that higher biofilm volume correlates positively with increased current production, the hypothesis of Edel et al. (2021) about a possible new quorum‐sensing mechanism in *S. oneidensis* is of interest. The authors claim that riboflavin induces the expression of the gene encoding the ornithine decarboxylase SpeC (*speC*) in a concentration‐dependent manner. This overexpression leads to increased biofilm formation possibly through protein–protein interaction, which in turn results in an increased current density in BESs. By overexpressing *speC*, a 1.89‐fold increase in mean current density could be achieved. This study highlights that our knowledge especially concerning anaerobic biofilm formation and the underlying mechanisms and networks is still very incomplete, and that more research is required in this area.

Ng et al. (2021) pursued a rather different strategy. The introduction of an additional copy of the gene cluster necessary for riboflavin production (*ribADEHC*) into *S. oneidensis* resulted in a 2.3‐fold increased final current density and 2‐fold increase in maximum power density. Although this is also interesting, in fact this strain generated by classical cloning was only a control for a rather different cloning approach. The authors aimed to introduce plasmids into already established non‐competent biofilms in situ by an ultrasound‐mediated DNA delivery technique. With this technique, a 1.6‐fold increase in final current density and a 2.8‐fold increase in flavin concentration for an already matured *S. oneidensis* biofilm in an MFC could be demonstrated compared to a wild‐type control group.

Focusing on the flavin expression as well, Yang et al. (2015) established a synthetic biosynthesis pathway for flavin from *Bacillus subtilis* in *S. oneidensis*, increasing flavin production and power density significantly. This endogenous flavin production could replace the addition of exogenous flavins in industrial applications and, thus, reduce process costs as flavins are rather expensive additives. Further, the authors postulate that not only outward EET but also inward EET were promoted with the addition of the synthetic flavin pathway. It is known that EAM including *S. oneidensis* and *G. sulfurreducens* among others perform EET in a bidirectional manner, which means that electrons can not only be transferred to an external electron acceptor, but also that electrons can be accepted by the organism via the same or a similar pathway. Inward EET has not been studied as intensively as outward EET in the context of application improvement; however, there are already several reports on this topic in the literature (Ross, Flynn, Baron, Gralnick, & Bond, 2011; Jiang & Zeng, 2019; Tefft & Teravest, 2019; Xie et al., 2021).

A more natural biofilm system in contrast to the model organism *S. oneidensis* was investigated by Yang et al. (2022), who pursued a strategy by directly utilizing a strain that exhibited higher current yields and biofilm formation a priori. Initially, the authors isolated a more efficient exoelectrogen from activated sludge, *S. carassii*‐D5, belonging to the genus *Shewanella*. Compared to *S. oneidensis*, *S. carassii‐*D5 showed a 5.4‐fold higher maximum power density. Furthermore, the level of *c*‐type cytochromes was increased and *S. carassii*‐D5 showed a stronger biofilm formation ability. The authors concluded that *S. carassii*‐D5 is pursuing an accelerated direct EET and aimed to increase indirect EET by introducing a synthetic gene cluster encoding the riboflavin synthesis pathway from *Bacillus subtilis* (*ribADEHC*). This resulted in a 4.7‐fold increase in riboflavin yields and a 1.6‐fold increase in maximum power output, which is a comparable improvement factor compared to the study by Ng et al. (2021).

Through the studies described above as well as through other more fundamental studies, we have already a rather complete picture on the mechanism of EET in *S. oneidensis*. Nevertheless, several questions remain. There is no clear answer as to what the specific function of OmcA in electron transfer is. Also, the genome of *S. oneidensis* contains the information for many cytochromes that could aid in electron transfer but which function is still unexplored. Overall, it is unclear why evolution has selected at all for multiheme proteins for the transfer of electrons from the cytoplasmic membrane to the cell surface. Along these lines, several attempts aimed at transferring the electron transport pathway from *S. oneidensis* to *E. coli*, but competitive electron transfer rates could not be achieved (see below). Hence, it is possible that some key functions for the process remain so far unexplored.

### Genetic optimizations for *Geobacter* species

**FIGURE 4:**
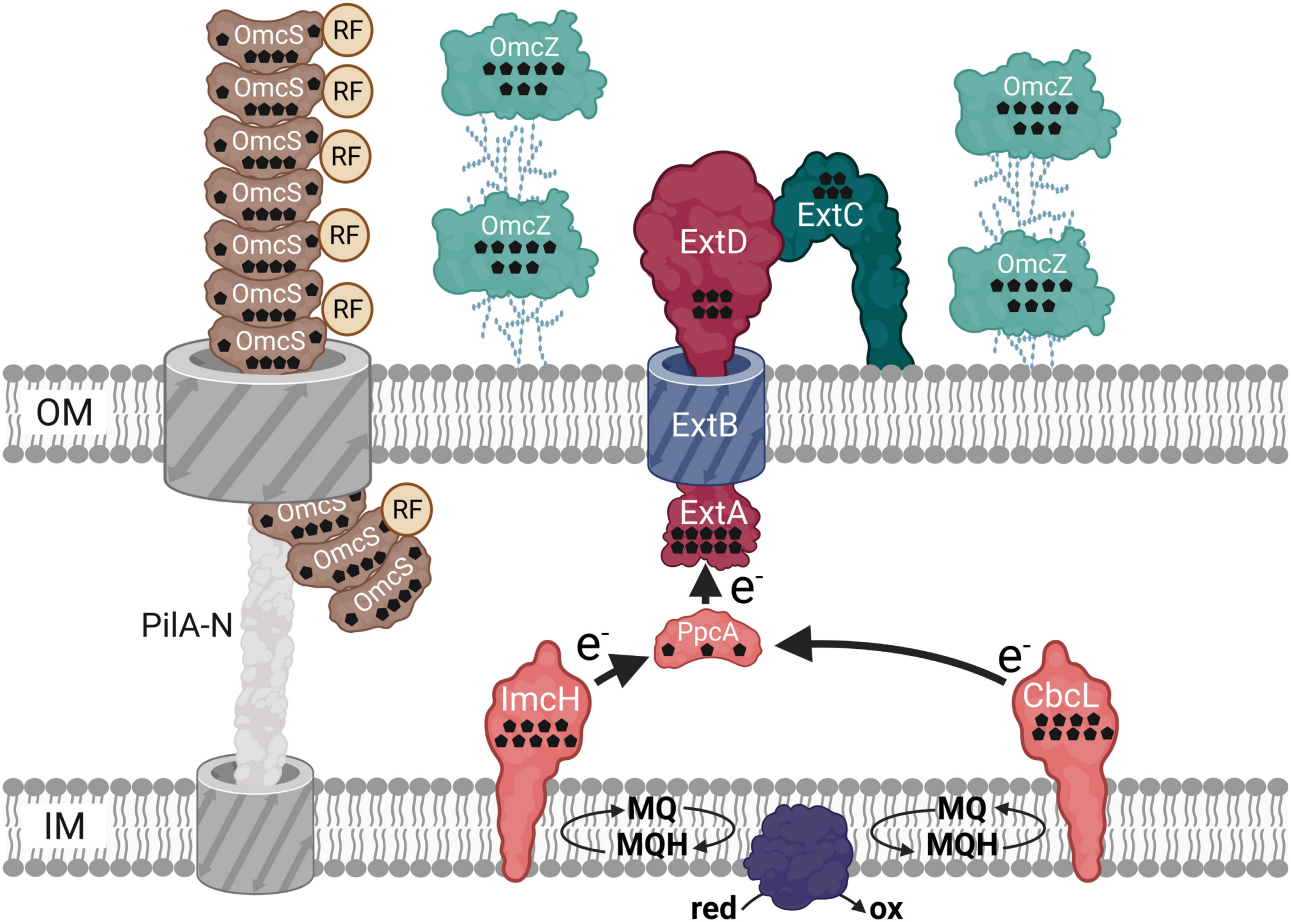
Scheme of the protein network for extracellular electron transfer (EET) in *G. sulfurreducens*. Black pentagons indicate the number of heme cofactors in *c*‐type cytochromes, IM stands for inner membrane, OM stands for outer membrane, the purple protein in the inner membrane represents an oxidoreductase and RF stands for riboflavin. Scheme modified from Edel et al. (2022).

Though most research groups rely on genetic modification of *S. oneidensis*, there are also studies reporting improved BES performance by genetically engineered *G. sulfurreducens* strains. As already described in detail for *S. oneidensis*, electron transfer in *G. sulfurreducens* is also mainly based on a network of *c*‐type cytochromes spanning the distance from the cytoplasmic membrane to the cell surface (see Figure 4). Although *Shewanella* and *Geobacter* species are very abundant in niches selecting for extracellular respiration, *Geobacter* biofilms in BES are generally characterized by catalysing higher current densities compared to *Shewanella* biofilms. The EET system in *Geobacter* species is more complex and extends far beyond the dimensions of single cells. So far, our knowledge is mainly based on the model organism *G. sulfurreducens*. Instead of only one element for electron transfer into the periplasm as in *S. oneidensis*, *G. sulfurreducens* uses either ImcH or CbcL for electron transfer into the periplasm. While CbcL is used when the terminal electron acceptor has a redox potential at or below −0.1 V versus SHE, ImcH is used for electron acceptors with a higher redox potential (Levar, Chan, Mehta‐Kolte, & Bond, 2014; Zacharoff, Chan, & Bond, 2016). Also, in *G. sulfurreducens*, electron transfer through the periplasm is mediated by a soluble *c*‐type cytochrome (Lloyd et al., 2003). At the outer membrane, various multimeric electron conduits can be used for electron transfer, but BES electron transfer relies mainly on ExtABCD (Otero et al., 2018). The most important difference in electron transfer strategies between the two organisms seems to be the extension of the electron transfer chain beyond the cell surface in *G. sulfurreducens*. Two strategies have been elucidated or are discussed to play a role. First, the octaheme *c*‐type cytochrome OmcZ was shown to be localized in the EPS of the organism (Rollefson, Stephen, Tien, & Bond, 2011). Moreover, the organism is able to build micrometre‐long conductive pili. The molecular reason for the conductivity of the pili remains controversial, although it has also been shown that these type IV pili are regularly decorated with *c*‐type cytochromes. Nevertheless, it was also elucidated that the *c*‐type cytochrome OmcS forms long‐protein filaments that can extend several micrometres away from the cell. In fact, there is evidence that these OmcS filaments are the nanowires that were previously thought to be formed by type IV pilus subunits and that were hypothesized to be conductive via overlapping π‐orbitals (Filman et al., 2019; Lovley and Walker, 2019; Wang et al., 2019; Wang, Hu, Dong, Shi, & Jiang, 2022). The export of these conductive structures is based on the type IV pilus production machinery, which might be the reason why they have been thought to be pilus subunits instead of cytochromes (Gu et al., 2021).

Leang, Malvankar, Franks, Nevin, and Lovley (2013) were able to generate a *G. sulfurreducens* mutant (CL‐1) through genetic engineering of the genome that resulted in a highly cohesive biofilm that was more conductive and produced 50% higher current density and 70% more power density than the wild‐type strain. GSU1240 was deleted, a gene encoding a protein with a PilZ domain, resulting in a massive overexpression of OmcS and PilA and the production of abundant exopolysaccharide not present in the wild‐type cells. Furthermore, CL‐1 produced biofilms that were 6‐fold more conductive than the wild‐type biofilm if grown with an electrode as the electron acceptor. This work is a good example that although *Geobacter* species already form very thick conductive biofilms currently producing the highest known current yields, the upper end of the performance of these organisms has probably not yet been reached. Furthermore, the possibility of light‐induced EET by the CL‐1 mutant was recently reported (Neu et al., 2022). Here, individual nanowires showed a 100‐fold increase in conductivity when photoexcited. With this, it would be possible to use sunlight for BES processes; however, the specific application procedure has to be further developed.

In a very recent study Wang et al. (2022) also followed a classical genetic engineering approach. The authors aimed to determine the applicability of nanowire proteins in improving bioelectricity production by overexpressing them. Overexpression of *pilA*, *omcZ*, *omcS* and *omcT* led to an increased voltage output of 1.32–1.63‐fold and an increased power density of 2.63–2.97‐fold.

Fujikawa, Ogura, Hayashi, and Inoue (2021); Fujikawa et al. (2021) did not follow the classical approach of targeted genetic manipulation but have attempted to isolate new strains or species with improved traits, which corresponds to the previously mentioned isolation of a *Shewanella* strain conducting higher electron transfer rates. With this approach, nine *G. sulfurreducens* strains were isolated from biofilms formed on an anode in a BES in which river sediment was used as an inoculum. One of the isolates, strain YM18, showed a 1.62‐fold increased maximum current density. Not the improvement itself, but the genomic differences of this strain compared to the type strain PCA are particularly interesting, as following this approach could lead to the identification of targets for future genomic modifications. For instance, surprisingly, *omcB*, *xapD*, *spc* and *ompJ*, which are known to be important genes for iron reduction and current production in PCA (Afkar, Reguera, Schiffer, & Lovley, 2005; Leang, Coppi, & Lovley, 2003; Liu, Zhan, Jing, Zhou, & Lovley, 2019; Rollefson et al., 2011), were not present in YM18. This indicates a genetic variation in key components involved in EET among different *G. sulfurreducens* strains. Moreover, this finding reemphasizes that our knowledge of EET is still incomplete, and further research is needed to fill the knowledge gaps. Along these lines, several open‐research questions mentioned in the previous section for *S. oneidensis* remain for the model organism *G. sulfurreducens* as well. The quantity of *c*‐type cytochrome encoding genes in this organism is even higher, and we are far away from understanding why evolution selected for these multitude and under which conditions their presence is accompanied with a specific fitness benefit. Also, here and in *S. oneidensis*, we have not understood the molecular processed and regulatory events that lead to anaerobic biofilm formation and how regulatory cues could steer the biofilm characteristics of the strains.

### Genetic optimizations for Gram‐positive EAMs

Although we have already stated that most scientists apply *Shewanella* and *Geobacter* species as model organisms for anodic BES research, multiple other EAMs capable of EET in BES have been identified and are reviewed elsewhere (Koch & Harnisch, 2016; Logan, 2009b; Logan, Rossi, Ragab, & Saikaly, 2019). Among those multiple Gram‐positive organisms have been shown to be capable of EET (Paquete, 2020). These include the pathogen *Listeria monocytogenes*, in which an eight‐gene locus was found to be associated with flavoprotein‐based EET processes and a simple strategy to build an electron conduit in Gram‐positive organisms was postulated (Light et al., 2018). Orthologues of this EET system have been found in various organisms, including for example *Entercoccus faecalis* (Hederstedt, Gorton, & Pankratovab, 2020) and a thermophilic *Geobacillus* strain (Shrestha et al., 2020).

For example, Zhang and colleagues reached impressive power densities above 400 mW m^−2^ utilizing the organism *Klebsiella pneumoniae* (Zhang et al., 2008). The organism was further investigated together with *S. oneidensis* in a synthetic consortium to broaden the substrate spectrum. (Li et al., 2018). The chosen electron donor was glycerol which was converted by *K. pneumoniae* to lactate, the preferred electron source for *S. oneidensis*. To expand the spectrum of usable carbon sources and to continuously provide lactate, Li and colleagues eliminated the ethanol pathway by the deletion of the native alcohol dehydrogenase in *K. pneumoniae* and further introduced a lactate dehydrogenase from *Lactobacillus bulgaricus* and a lactate transporter from *E. coli*. These genetic modifications led to a power density of about 19.9 mW m^−2^, which is a 2.44‐fold increase in comparison with the native consortium. As only one strain was genetically engineered, this study shows a different approach on how to improve performance and demonstrates the multiple aspects that should be taken into consideration when improving BESs. In comparison with the common model organisms *S. oneidensis* and *G. sulfurreducens*, the application of various other EAMs was neglected in the past due to the lower productivity in terms of power generation. Nevertheless, studying these weak electricigens could boost the understanding of EET process and further open up the possibility of improving substrate spectra and application sites (Doyle & Marsili, 2018).

## OPTIMIZATION OF THE BACTERIA–ELECTRODE INTERFACE AT THE ABIOTIC LEVEL

A completely different approach is the pretreatment of bacteria or modification of the electrode with various chemical, nano‐ or biopolymeric compounds, while the genetic background of the studied organisms and also the organisms themselves are not altered. The main goal here is also to improve current production or energy yields. Similar to the genetic approach, the abiotic approach targets to be improved include optimized adhesion, changes in conductivity of the EPS matrix, increased biofilm formation as well as secretion of shuttle molecules such as riboflavin (Du, Catania, & Bazan, 2014; Glaven, 2019; Guo, Prévoteau, Patil, & Rabaey, 2015; Zhao et al., 2021). In the following, recently reported technical improvements are discussed, and it will be distinguished between pretreatment of bacteria and modification of the electrode material.

### Pretreatment and coating of anodic biofilms

The pretreatment of cells can further be categorized in the coating of organisms with chemical or biological compounds and the embedding of bacteria in polymeric structures. The latter provides the organisms with a 3D network that is often conductive and therefore allows optimized positioning of cells between the stationary electron acceptor and the electron donor, which is dissolved in the electrolyte and affected by mass transfer limitations (Renslow et al., 2013). Applied conductive polymeric matrices often directly increase the surface area of the electrode and are acting as current collector being in direct contact with the electrode and the cells (Oh & Logan, 2006; Sonawane, Yadav, Ghosh, & Adeloju, 2017). Pretreatment with chemical compounds or nanostructures, on the other hand, often alters the cell surface as well as the EPS matrix properties of the biofilm. Besides the influences on the structural properties, nanomaterials are known to alter bacterial metabolism, which can lead to an increased EET compared to unmodified biofilms (Chen et al., 2018; Savla, Anand, Pandit, & Prasad, 2020). Often, cell coating results in increased EPS conductivity and/or formation, as well as increased secretion of self‐produced electron shuttles by the organisms. In the end, both strategies lead to the assembly of hybrid biocomposites that can lead to an improved performance in BES applications.

Embedding bacteria in conductive polymers was investigated in an interesting approach by Zajdel et al. (2018). The authors used poly‐3,4‐ethylenedioxythiophene::poly‐styrenesulfonate (PEDOT:PSS) as 3D matrix for *S. oneidensis* biofilms. This resulted in a 20‐fold increase in steady‐state current. The organisms were mixed with the toxic monomer during electropolymerization and their vitality maintained, which enabled their application in an MFC. While most previous studies focused on increasing electrode surface area or cell attachment through electrode treatment, the authors postulate that they connected more cells via the 3D biocomposite to the electrode, resulting in higher performance. Based on this biomaterial, multiple application strategies emerged that allowed for easier implementation of the bacteria–hydrogel composite. For example, Freyman, Kou, Wang, and Li (2019) demonstrated that it was possible to 3D‐print an anode from alginate hydrogel and *S. oneidensis* cells. However, no negative control was performed, so no improvement factor can be calculated for this study. Consistent with this embedding into conductive polymers, Knoll, Fuderer, and Gescher (2022) used agarose hydrogels containing riboflavin‐functionalized carbon nanofibres, which resulted in a 9‐fold increase in mean current density and demonstrated that it was even possible to spray this biomaterial onto the electrode surface. This was the first report on spraying biofilm material, and the latter could facilitate the application of biocomposites in industrial processes. Further, McCuskey, Su, Leifert, Moreland, and Bazan (2020) reported the self‐assembly of a biocomposite of *S. oneidensis* cells and the polyelectrolyte 2,6‐(4,4‐bis‐potassium butanylsulfonate‐4H‐cyclopenta‐[2,1‐b;3,4‐b']‐dithiophene)‐alt‐4,7‐(2,1,3‐benzothiadiazole) (CPE‐K) onto a gold electrode that resulted in a 150‐fold increase in the current density. Self‐assembly describes the process in which single components of a system such as molecules, polymers or macroscopic particles order themselves in a structured and/or functional way due to specific interaction between these components without external direction (Varga, 2016). This specific biocomposite from CPE‐K and *S. oneidensis* cells was also studied regarding the interaction with commercially available carbon paper rather than gold electrodes, which might allow for an industrial application (Vázquez et al., 2022). Here, the developed synthetic biofilm catalysed only a 6‐fold increased electron transfer.

With regard to single‐cell treatment rather than embedding of cells, Arinda et al. (2019) demonstrated that preincubation of *S. oneidensis* with riboflavin‐coupled magnetic beads resulted in a 6.5‐fold improvement in mean current density. However, the authors postulate that the beneficial effect of riboflavin is not necessarily due to its role as cofactor or electron shuttle but showed that the presence of riboflavin promotes biofilm formation, which was later linked to *speC* by Edel et al. (2021). Yu et al. (2020) went one step further by constructing *S. oneidensis* cells as unicellular current collectors, improving the connectivity of electron transfer pathways. They polymerized either polydopamine or synthesized FeS nanoparticles in situ on individual cells, with FeS treatment leading to higher improved performance. The polydopamine and especially the nanoparticles connect the inner membrane electron pool of the cells to the external electron collectors, leading to an improvement in maximum power density by up to 16‐fold. Regarding the effects of polydopamine, Wang et al. (2021) used this polymer in combination with polypyrrole to coat *S. oneidensis* cells, which led to a 12‐fold improvement in maximum power density. This effect was related to an increase in riboflavin secretion that was measured for the coated cells in comparison with the non‐modified organisms. Using *Shewanella putrefaciens* as a test organism, Sharma, Li, Hu, Chang, and Yu (2021) immobilized the cells in a polyvinyl alcohol/sodium alginate hydrogel spiked with graphite nanoparticles and anthraquinone‐2,6‐disulfonate (AQDS), leading to a 1.6‐fold increase in terms of maximum power density. AQDS is added here to provide a soluble electron shuttle that allows indirect electron transfer, whereas the graphite nanoparticles provide conductivity and thus DET throughout the hydrogel.

So far, only studies with pure cultures have been mentioned. Studies with pure cultures offer the advantages that a simplified model can be applied and that it is easier to compare between optimizations. However, the application of MFCs, e.g., in wastewater treatment, often employs biofilms of complex mixed cultures derived from anaerobic sludge. In the following, these mixed cultures were embedded or treated before their implementation in MFCs. For example, Massaglia, Sacco, Chiodoni, Pirri, and Quaglio (2021) embedded a mixed culture directly into nanofibres of polyethylene oxide formed during the electrospinning process. The cells were mixed with polyethylene oxide prior to electrospinning. This resulted in a 2‐fold increase in performance. As a control, cells were planktonically inoculated in a single‐chamber MFC in which polyethylene oxide without cells was electrospun as electrode. Besides this, Li et al. (2021) nano‐decorated a mixed culture with self‐assembled gold nanoparticles as well as reduced graphene oxide (rGO). rGO is already known to stimulate EET at the microbe–electrode interface (Yong, Yu, Zhang, Song, 2014). The power density of the modified biofilm increased 1.4‐fold.

### Modification of the anode material

Besides coating or embedding of the biofilm organisms, the interface between bacteria and electrode can be improved by electrode modifications. Several studies have been conducted on the optimization of electrode materials within the last decade (Hindatu, Annuar, & Gumel, 2017; Kumar, Sarathi, & Nahm, 2013; Li, Cheng, & Thomas, 2017; Nosek, Jachimowicz, & Cydzik‐Kwiatkowska, 2020; Park et al., 2022); thus, in this work a comparable overview of the most recent advances and the involved new technologies is given so that the combination of improvement strategies can be discussed later. When choosing electrode materials, it can be broadly categorized between carbon‐based and metal‐based materials, both of which have opposite advantageous properties (Guo et al., 2015). Carbon‐based electrodes offer higher biocompatibility and are often less expensive and more corrosion resistant. However, electrical conductivity and mechanical strength must be optimized for these materials to be used on a large scale. In contrast, metal‐based electrodes are not particularly biocompatible but offer better conductivity and mechanical strength. In addition, the costs of these materials are expected to be high, further hindering their industrially relevant use. The optimizations addressed (Table 2) almost exclusively examine biocomposites – the combination of metal‐ and carbon‐based materials. These biocomposites combine the beneficial properties of both materials and balance out the negative properties.

**TABLE 2:** Comparison of approaches for improving biofilm–electrode interaction at the abiotic level. Improvement factor (IF) in regard to a = steady‐state current (stable current), b = mean current density (integrated current × area^−1^ × time^−1^), c = current density (current × area^−1^), d = average current (current × time^−1^), e = current per cell (current × cell number^−1^), f = maximum power density (voltage × internal resistance^−1^ / power × area^−1^) and e = maximum electric energy (integrated power density). In each case, the corresponding absolute value is given in parentheses. SS refers to stainless steel.

Organism	Improvement strategy	Anode	IF	Reference
Coating/Embedding of cells				
*Shewanella oneidensis*	Cells embedded in PEDOT:PSS	Carbon felt	20×^a^ (30 μA)	Zajdel et al. (2018)
	Binding of cells to magnetic riboflavin‐beads	Carbon felt	6.5×^b^ (0.2 A m^−2^)	Arinda et al. (2019)
	Single‐cell current collectors by in situ polymerization of polydopamine or synthesis of FeS nanoparticles on individual cells	Carbon felt	1.6–1.9×^e^ (0.2 mA m^−2^) 3.3 – 16×^f^ (3.2 W m^−2^)	Yu et al. (2020)
	Coating of cells with polypyrrole and polydopamine	Carbon felt	12×^f^ (2.5 W m^−2^)	Wang, Pan, et al. (2021)
	Cells embedded in agarose hydrogels spiked with carbon nanotubes and riboflavin sprayed on electrode	Carbon felt	9.1×^b^ (1.3 A m^−2^)	Knoll et al. (2022)
	Self‐assembly of composite of cells and conjugated polyelectrolyte CPE‑K for immobilization on electrode	Carbon paper	5.7×^c^ (0.3 A m^−2^)	Vázquez et al. (2022)
	Self‐assembly of composite of cells and conjugated polyelectrolyte CPE‑K for immobilization on electrode	Gold silicon wafer	151×^c^ (0.2 A m^−2^)	McCuskey et al. (2020)
*Shewanella putrefaciens*	Immobilization of cells with polyvinyl alcohol/sodium alginate hydrogel spiked with graphite nanoparticles and AQDS as mediator	Graphite felt	1.6×^f^ (4.1 W m^−3^)	Sharma et al. (2021)
Mixed culture	Embedding of cells into electrospun nanofibres based on polyethylene oxide (PEO) as polymeric matrix	Carbon paper	2×^f^ (0.3 A m^−2^)	Massaglia et al. (2021)
	Modification of biofilm by addition of gold nanoparticles and reduced graphene oxide (rGO)	Graphite felt	1.4×^f^ (0.6 W m^−2^)	Li, Sun, Tang, Zhou, et al. (2021)
Modified electrode				
*Shewanella oneidensis*	Coating with modified agarose hydrogels spiked with gold nanoparticles	Carbon veil	18×^d^ (33.6 μA)	Suravaram et al. (2019)
	Polypyrrole‐carboxymethyl cellulose‐titanium nitride carbon brush hydrogels as bioanode	Carbon brush	4.7×^f^ (14.1 W m^−3^)	Wang et al. (2020)
	Modification with rGO and silver nanoparticles	Carbon paper	15×^c^ (9.2 A m^−2^) 13×^f^ (6.6 W m^−2^)	Cao et al. (2021)
	Coating with PEDOT:PSS/PHEA and polydopamine	Indium tin oxide	178×^c^ (0.6 A m^−2^)	Tseng et al. (2022)
*Shewanella loihica*	TiO_2_‐TiN nanocomposite for hybrid biofilm	Carbon paper	5.6×^c^ (0.3 A m^−2^)	Su et al. (2020)
	Modification with synthesized α‐FeOOH nanowires	Carbon paper	3.2×^f^ (0.1 W m^−2^)	Xian et al. (2021)
*Rhodobacter spaeroides*	Modification with vitamin‐C‐enabled rGO for biofilm for methane/methanol treatment in MFCs	Nickel foam	540×^c^ (0.7 A m^−2^) 220×^f^ (1.2 W m^−2^)	Islam et al. (2020)
Mixed culture	Surface modification with polydopamine and rGO	Carbon cloth	6.1×^f^ (2.0 W m^−2^)	Li et al. (2020)
	Modification with graphene/Fe_2_O_3_ composite	Carbon felt	2.6×^f^ (0.3 W m^−2^)	Fu et al. (2020)
	Loading with graphene, graphene oxide or carbon nanotubes for sediment MFC	Carbon felt	1.1–2×^e^ (0.98 kJ)	Liang, Zhai, Liu, Ji, and Li (2020)
	Modification with magnesium oxide of 3D‐electrode	Biochar	1.6×^c^ (14 A m^−3^) 1.8×^f^ (4.5 W m^−3^)	Dong et al. (2020)
	Aerogel anode integrated with cerium dioxide (CeO_2_) nanotubes decorated nitrogen‐doped reduced graphene oxide nanosheets (NRGO)	3D Carbon aerogel	9.7×^f^ (1.5 W m^−2^)	Senthilkumar et al. (2020)
	Modification with polyaniline‐functionalized activated carbon	SS mesh	1.9×^f^ (0.3 W m^−2^)	Yellappa, Annie Modestra, Rami Reddy, and Venkata Mohan (2021)
	Coating with composite of polydimethysiloxane (PDMS) spiked with carbon nanofibres	SS mesh	5.1×^f^ (0.02 W m^−2^)	Saadi et al. (2020)
	Coating with composite of polydimethylsiloxane (PDMS) spiked with carbon nanofibre	Cupper foil	8.7×^f^ (0.07 W m^−2^)	Bensalah et al. (2021)
	Surface modification using iron phthalocyanine	Carbon cloth	4.3×^f^ (2.5 W m^−2^)	Li, Liu, Chen, Yuan, et al. (2021)
Modified electrode				
	Fabrication of manganese cobalt oxide anode using a conventional carbon felt	Carbon felt	7.7×^c^ (1.2 A m^−2^) 3.8×^f^ (0.9 W m^−2^)	Tahir et al. (2021a)
Mixed culture	Coating of carbon felt electrode with nickel ferrite/Mxene	Carbon felt	6.6×^c^ (1.0 A m^−2^) 5.6×^f^ (1.4 W m^−2^)	Tahir et al. (2021b)
	Modification with L‐threonine or PANDAN	Carbon felt	1.3–1.9×^f^ (0.04 W m^−2^)	Li et al. (2021)
	Co‐modification with iron porphyrin and polyquaternium‐7	Carbon cloth	1.9×^f^ (2.2 W m^−2^)	Li, Liu, Chen, Wu, et al. (2021)
	Modification with nitrogen‐doped carbon quantum dots decorated with iron/iron oxide nanoparticles	Carbon cloth	4.0×^c^ (2.6 A m^−2^) 3.3×^f^ (0.8 W m^−2^)	Habibi et al. (2021)
	3D nanostructures electrode from nitrogen‐doped carbon and ferrite	Carbon cloth	2.1×^f^ (1.2 W m^−2^)	Wang, Liu, et al. (2021)
	Modification with tungsten carbide and rGO	Carbon felt	3.6×^c^ (5.7 A m^−2^) 4.4×^f^ (1.1 W m^−2^)	Mohamed et al. (2021)
	Modification with *β*‐cyclodextrin and polydopamine	Carbon felt	1.9×^c^ (0.2 A m^−2^) 4.6×^f^ (0.1 W m^−2^)	Fan and Xi (2022)
	Hybrid electrode modified with Fe_4_Co	Carbon cloth	1.7×^f^ (2.0 W m^−2^)	Chen et al. (2022)
	Deposition of candle soot‐derived carbon nanoparticles	SS mesh Titanium mesh	18×^f^ (0.4 W m^−2^) 986x^f^ (0.6 W m^−2^)	Pu et al. (2022)
	Modification with composite of MnO_2_ and multiwalled carbon nanotubes	Silver mesh	112×^f^ (0.4 W m^−2^)	Mahmoud et al. (2021)

For carbon‐based materials, Suravaram, Smith, Parkin, and Chechik (2019) used an agarose gel spiked with gold nanoparticles to improve the average current about 18‐fold utilizing *S. oneidensis* cells. The authors modified the agarose with 2‐chloroethylamine to introduce more gold‐binding amine groups to the gel which led to an even distribution of gold nanoparticles, and therefore a higher surface area as well as a better availability of electron acceptor due to an increase in conductivity of the gel itself. Further, polypyrrole is another conductive polymer that has been applied for electrode modification in MFCs due to its high conductivity and good environmental stability (Chi, He, Wang, Zhou, & Gu, 2013; Feng et al., 2010). However, this polymer has a low solubility which limits ion adsorption and charge transfer. Therefore Wang, Wen, Chen, and Li (2020) designed a composite from polypyrrole, carboxymethyl cellulose (CMC) and titanium nitride (TiN). As a highly conductive coating material, TiN was until now only applied in lithium‐ion battery research (Snyder et al., 2007). As a water‐based binder for this three‐component composite, CMC exhibited biocompatibility and nontoxicity. Overall, a 5‐fold increase in power density due to a larger surface area was achieved (Wang et al., 2020). Additionally, TiN was used to form a nanocomposite together with TiO_2_ as well (Su, Yin, Du, Zhang, & Fu, 2020). The beneficial effects of TiO_2_ on flavin secretion (Maurer‐Jones, Gunsolus, Murphy, & Haynes, 2013) and electron transfer rates (Yin, Lin, Su, Yuan, & Fu, 2015) together with TiN led to a 5.6‐fold increase in current density in an MFC utilizing *Shewanella loihica* cells.

Cao et al. (2021) showed in an impressive study that rGO combined with silver nanoparticles can boost power density produced by *S. oneidensis* with a factor of 13 to about 6.6 W m^−2^. The authors state that with their method, they surpassed the power limit of 0.3 W m^−2^ of current MFCs due to the fact that their electrode was designed in a manner that extracted metabolic electrons in an optimized way. Silver nanoparticles and rGO led to an increase in biofilm formation, and by determining the cell number of the biofilms, an individual turnover frequency per cell was given. The resulting analysis revealed that charge transfer processes have indeed improved. Scanning transmission electron microscopy (STEM) and energy‐dispersive X‐ray spectroscopy (EDX) were further applied to analyse the membrane of individual cells, and silver nanoparticles were shown to be located inside and across the membrane. The authors postulate that the silver ions were released by the electrode and were in situ reduced by the cells. These formed nanoparticles built a metallic shortcut, explaining the improved performance of those *Shewanella–*silver hybrids (Cao et al., 2021). As another nanomaterial‐based improvement, α‐FeOOH nanowires were synthesized on carbon paper, which increased power density by 3‐fold in an MFC with *S. loihica* (Xian et al., 2021). These nanowires decreased electron transfer resistance while increasing biofilm formation and EET, simultaneously.

For mixed culture studies, carbon‐based electrodes have been improved with rGO and polydopamine (Li et al., 2020). Both of these compounds' advantageous properties have already been mentioned, but in this combination, polydopamine provides hydrophilicity and superior adhesive force, while rGO offers electrochemically active sites for improved electron transfer, resulting in a 6‐fold increase in power density to about 2 W m^−2^. Further, Fan and Xi (2022) combined polydopamine with *β*‐cyclodextrin for 4.6‐fold increase in power density. *β*‐cyclodextrin affects fluidity and permeability of biofilms by inducing lipid exchange between cells (Hammoud et al., 2019). Another method for optimizing electrode properties is the use of heteroatom doping or coating. For example, nitrogen doping can enhance bacterial attachment by providing more electroactive sites for EET processes (Cheng & Logan, 2007; Wang et al., 2009). Li et al. (2021) utilized iron porphyrin combined with polyquanternium‐7 for an improved power density of 2.2 W m^−2^ (2‐fold increase). Porphyrins are abundant in nitrogen atoms, so a nitrogen‐doped electrode was provided. As iron porphyrin tends to form aggregates, Li, Liu, Chen, Wu, et al. (2021) combined it with polyquanternium‐7, a polymer to increase hydrophilicity of this biocomposite. In addition, the compound iron phthalocyanine has a similar structure to iron porphyrin and was used to modify a carbon cloth which led to an improved power density of about 4‐fold to 2.5 W m^−2^ (Li et al., 2021). Regarding nitrogen‐doped material, Habibi, Arvand, and Sohrabnezhad (2021) optimized carbon cloth with nitrogen‐doped carbon quantum dots in combination with iron/iron oxide nanoparticles. Interestingly, this is the first known use of carbon quantum dots in MFCs. In this application, the quantum dots anchored the nanoparticles while improving conductivity and surface area. The combination of nanoparticles and quantum dots led to a 3.3‐fold improvement in power density.

Additionally, ferrite (Fe_2_O_3_) as a compound for electrode modification has been studied in multiple cases. The iron oxide has a high affinity to *c*‐type cytochromes as shown for *Shewanella* cells (Beliaev, Saffarini, McLaughlin, & Hunnicutt, 2001) and therefore increases electrical output of cells. Fu, Wang, Huang, Song, and Xie (2020) combined ferrite with graphene for a 2.6‐fold increase to 0.3 W m^−2^ in power density, and Wang et al. (2021) used the aforementioned nitrogen‐doped carbon with ferrite for an improvement of 2.1‐fold to 1.2 W m^−2^. Further, Tahir et al. (2021b) utilized nickel ferrite which led to superior electrocatalytic activity due to the presence of nickel and iron ions. A third part of this biocomposite was MXene, a material that belongs to a new family of two‐dimensional metal carbide nanosheets that are similar to graphene (Anasori, Lukatskaya, & Gogotsi, 2017). Accordingly, a power density of 1.4 W m^−2^ was achieved, which is the highest among the three studies presented here utilizing ferrite for optimization. Apart from this, tungsten carbide has been studied as an additive due to its high metallic conductivity, corrosion resistance and long‐term durability (Niu, 2018). However, the low wettability and the complexity of its fabrication hinder application. Thus, Mohamed et al. (2021) combined tungsten carbide with rGO and achieved an improved power density of 1.1 W m^−2^, which is a 4.4‐fold improvement. In addition, Chen et al. (2022) studied the effect of a hybrid electrode from carbon cloth and Fe_4_Co. The beneficial effects of iron ions have already been discussed, while cobalt ions are known to act as a growth inhibitor. The authors performed a live/dead staining, and a viability value was calculated based on the ratio of viable to total cells. Fe_4_Co led to an activation of the inner layer of the biofilm by stimulating the secretion of EPS components, and thereby a highly active biofilm was formed. Overall, a power density of 2 W m^−2^ was achieved, which corresponds to a 1.7‐fold increase compared to the unmodified carbon cloth.

Electrode materials can be two‐ or three‐dimensional, whereas the latter provides beneficial effects for bacterial attachment (Zou et al., 2016). Often, pre‐cursor materials are used to provide complex 3D structures. One of those pre‐cursors is biochar, a waste product from biomass processes that has already been applied as electrode material before (Huggins, Wang, Kearns, Jenkins, & Ren, 2014). Magnesium oxide was used to modify the surface of a biochar‐based 3D electrode (Dong, Wu, Wang, Lu, & Li, 2020), and thereby the introduction of metal ions such as magnesium improved ion exchange capacity and provided more adsorption sites (Zhang, Gao, Yao, Xue, & Inyang, 2012). The study of Dong et al. (2020) further focused on these 3D anodes for chemical oxygen demand (COD) and nitrogen removal in wastewater. Treatment with magnesium oxide led to a 1.8‐fold improvement in power density to 4.5 W m^−3^ and the nitrogen removal rate increased by 1.2‐fold to about 85%. Concerning 3D electrodes, Senthilkumar et al. (2020) designed a 3D carbon aerogel electrode derived from waste paper. The authors employed cerium oxide (CeO_2_) for the modification, and the nearly tenfold improvement in power density to 1.5 W m^−2^ was explained by the biocompatibility as well as the superior capacitive properties of CeO_2_ (Maheswari & Muralidharan, 2016). Moreover, it is stated that CeO_2_ is known to transfer electrons directly from the cells to the electrode surface due to hydrogen bonds formed with the positively charged lysine residues of *c*‐type cytochromes (Qu, Dong, Cheng, Lu, & Dong, 1996). However, CeO_2_ is a semiconductor; thus, the authors compensated for the poor electrical conductivity by using nitrogen‐doped rGO nanosheets as support material, for which the beneficial effects of nitrogen doping and rGO have already been discussed in this review.

Regarding metal‐based modifications, Tseng et al. (2022) modified an indium tin oxide (ITO) electrode with PEDOT: PSS, 2‐hydroxyethylacrylate (PHEA) and polydopamine. The latter was used as a thin layer for adhesion to the ITO‐coated glass of the combined mixture of PEDOT:PSS and PHEA. PEDOT:PSS as conductive polymer has already been discussed above. However, the authors found that the addition of PHEA stabilized the biocomposite and in their BES with *S. oneidensis* cells, the modified ITO electrode resulted in a 178‐fold improvement in current density to 0.6 A m^−2^. That improvement value is higher than previously reported for 3D coating with conductive polymer coatings. Further, Islam et al. (2020) coated a 3D nickel foam electrode with multiple rGO layers. The study focused on methane/methanol treatment in MFCs using *Rhodobacter sphaeroides* a previously unexplored methylotroph, and the surface modification led to altered surface properties that improved biofilm growth of the organism. Interestingly, for the deposition of the multiple layers of rGO, vitamin C was used as a reducing agent. The authors presented a cost analysis showing that their modification method resulted in an electrode that is 55 times cheaper than commercially available graphene/nickel electrodes. In total, a power density of 1.2 W m^−2^ was achieved, which corresponds to a 220‐fold improvement. Polydimethylsiloxane (PDMS) is a polymer, which was not discussed so far. This polymer was combined with carbon nanofibres for the improvement of copper (Bensalah et al., 2021) as well as stainless steel (Saadi et al., 2020) anodes. PDMS itself is known as an elastomer used for the fabrication of microfluidic systems. Conductivity could be introduced to this polymer by incorporating conductive nanoparticles, such as carbon nanofibres (Niu, Peng, Liu, Wen, & Sheng, 2007). Saadi et al. (2020) were the first to apply this composite to stainless steel electrodes in MFCs and achieved a 5.1‐fold improvement in power density to 0.02 W m^−2^. Coating a copper electrode with PDMS spiked with carbon nanofibres further improved power density to 0.07 W m^−2^, which represents an 8.7‐fold improvement (Bensalah et al., 2021). In addition, silver mesh was modified with manganese oxide (MnO_2_) and multiwalled carbon nanotubes (Mahmoud, Samhan, Ibrahim, Ali, & Hassan, 2021). Low cost, biocompatibility as well as the pseudo‐capacitive properties of MnO_2_ are beneficial characteristics of this compound, which, however, offers only a small surface area without a support material. To overcome this issue, MnO_2_ was deposited on multi‐walled carbon nanotubes, resulting in a 112‐fold increase in power density to about 0.4 W m^−2^. Moreover, Pu et al. (2022) used a rather impressive method to deposit carbon nanoparticles for the modification of titanium mesh, stainless steel and copper sheets. Carbon nanoparticles derived from candle soot were already utilized in MFCs on stainless steel electrodes by Singh, Bairagi, and Verma (2018), but the deposition led to uneven layers of carbon nanoparticles that easily detached from the modified material. Thus, Pu et al. (2022) used a series of flame deposition, surface paraffin combustion, and heat treatment to achieve uniform deposition of carbon nanoparticles. Flame deposition coated the material with carbon nanoparticles, while paraffin combustion bound the layer tightly to the surface. To improve hydrophilicity, the final step was heat treatment at 280 °C, and this three‐step modification resulted in a 1000‐fold improvement in power density to about 0.6 W m^−2^ for the titanium fabric, for example.

In conclusion, metal‐based modifications have been intensely studied in the literature and the power limit of 0.3 W m^−2^, long considered the limit (Cao et al., 2021), has been surpassed by several modification procedures presented in this review (Table 2). However, a combination of the most impressive strategies – including the combination of cell coating/embedding and electrode modification – could allow power densities to be pushed to even higher levels.

## FUTURE DIRECTION OF BES PERFORMANCE OPTIMIZATION

### Genetic engineering – Synthetic combination of electron transport chain components as well as biofilm formation as future targets

Genetic engineering of exoelectrogenic bacteria to increase current and energy yields contributes not only to improved performance but also to a fundamental understanding of EET processes. Considering the number of studies reporting on genetic optimizations, there are surprisingly few recent research items reporting increased current production or power densities through genetic engineering. The improvement factors for current and energy production range from 1.17 to 3.62 (see Table 1). Genetic modifications are mainly carried out in *Shewanella* most likely because of its genetic accessibility. In this mini‐review, we were mainly concentrating on recent publications with a focus on improvements of the bacteria–electrode interface, but the observation is also in line with previous publications (Philipp, Edel, & Gescher, 2020). Evidently, major improvements in MES technology are rarely achieved through genetic modification. It can be speculated that the metabolic ability to exploit extracellular electron acceptors has been optimized during evolution in a way that electron transfer cannot be accelerated much further even if conducted in a synthetic environment such as a BES. Nevertheless, at least the comparison of *Geobacter* and *Shewanella* species reveals that there is potential for improving electron transfer by advancing the biological systems. BES systems operated with *Geobacter* species are generally characterized by considerably higher current densities compared to systems relying on *Shewanella* cells. This seems to be mostly due to the strategy of *Geobacter* species to extend their electron transfer machinery into the biofilm EPS using conductive wire‐like structures and sugar‐polymer embedded cytochromes. It should at least be possible to copy that strategy into *Shewanella* cells. Along these lines, it might be worth considering to pursue a full synthetic biology approach in which parts from different EAMs are synergistically combined within one chassis organism. So far, this approach was only pursued to transplant the electron transfer chain from *S. oneidensis* into *E. coli*. Although this approach was to some extent successful, it was not possible to reach EET rates in *E. coli* that would be similar to what is reached by *Shewanella* cells (Beblawy et al., 2020; Jensen et al., 2010; Sturm‐Richter et al., 2015; TerAvest, Zajdel, & Ajo‐Franklin, 2014). Hence, we have apparently not understood the biochemistry sufficiently to transplant it in way reaching its full capacity in other organisms yet.

As stated above, a target for further genetic improvement of naturally exoelectrogenic bacteria could be biofilm formation. By increasing the maximum biofilm thickness, higher current densities per electrode area could be achieved biologically, although mass transfer limitation and conductivity across the biofilm is likely to be a problem. This is in line with the findings of Malvankar et al. (2012) in which the authors correlate conductivity of biofilms directly with current production. In this context, Torres, Marcus, and Rittmann (2008) already hypothesized that proton transport out of the biofilm limits electrical current generation. While there are accepted theories about the formation of biofilms and a large number of reviews have already been published in this context (Flemming et al., 2016; Flemming & Wingender, 2010; O'Toole, Kaplan, & Kolter, 2003; Rather, Gupta, & Mandal, 2021; Tolker‐Nielsen, 2015), this research has been conducted primarily with oxic biofilms (Kragh et al., 2016; Lawrence, Korber, Hoyle, Costerton, & Caldwell, 1991; Liu et al., 2015; O'Toole & Kolter, 1998; Sauer, Camper, Ehrlich, Costerton, & Davies, 2002). It appears that the fundamentals of anoxic biofilm formation, especially compared to oxic biofilm formation, are poorly studied and therefore poorly understood. Fundamental research will help to understand underlying mechanisms and thus identify targets for genetic improvements.

### New electroactive organisms will broaden our toolbox to engineer future electroactive organisms

Concerning electroactive bacteria, researchers have mainly focused on model organisms such as *Shewanella* and *Geobacter* species to understand the fundamentals of EET, and therefore the comparison in this review naturally focuses on these model organisms. However, Light et al. (2018), Santoro et al. (2021) as well as Edel, Philipp, Lapp, Reiner, and Gescher (2022) have shown that there is an enormous number of organisms that perform EET based on flavins or *c*‐type cytochromes that have not been considered in BES applications yet. The diversity of these unknown metabolic pathways and microorganisms could be the foundation for advanced BES applications. Again, a better understanding of these fundamentals could help identifying targets for genetic improvement and development of synthetic pathways operating in model organisms. Furthermore, as different organisms could optimize different areas within the electroactive biofilm of BES processes, new application opportunities arise due to differences in substrate spectra, tolerance to extreme environmental conditions and EET efficiencies. This is highlighted in the study by Li et al. (2018), where optimization of the glycerol metabolism of *K. pneumoniae* improved the interaction with *S. oneidensis*.

Genetic modification could be a major challenge in the future. Since the genetic accessibility of each organism varies greatly, tools for modifying the genetic background of these organisms must first be developed. Recent advances for genetic engineering of EAMs are summarized in a review by Bird et al. (2021). This review focuses not only on the established tools for engineering native EAMs, but further gives an overview of the introduction of EET pathways into *E. coli*. For a better understanding of the EET processes in general, this extension of the genetic toolbox is a promising aspect, and the deeper insight into the molecular mechanisms of EAM could be another breakthrough for the application of MES.

### Seeing the biofilm–electrode interaction as one biohybrid material offers possibilities for larger improvements in current density

Compared to biotic improvements, there is a plethora of recent publications that report improved current and energy yields through improvement of the bacteria–electrode interface at the abiotic level. The improvement factors here range from 1.1‐ to 1000‐fold, and the modifications carried out are of a very different character (see Table 2). The highest improvement rates could be achieved by improving different non‐carbon‐based metal electrodes. While it is possible to achieve far greater improvements with non‐carbon‐based electrodes, the use of more costly metal‐based electrodes seems more practical for small‐scale research or biosensors, where the costs and benefits are balanced by the small size. For large‐scale applications, such as wastewater treatment or electrode‐assisted fermentation, carbon is the standard material because it is both much cheaper and more widely available. We have already speculated about the evolutionary adaptation of exoelectrogenic bacteria. These microorganisms have only been able to adapt to naturally occurring materials. Therefore, it is reasonable to assume that the development of new materials will give rise to an entire new field of interaction possibilities. The development of new materials can thus be understood as the creation of new ecological niches. From this perspective, it is not surprising that much greater improvement factors can be achieved by technical means than by genetic means.

Besides the high improvement factors achieved by technical approaches, it is clear that a combination of electrode material modification and coating or embedding of single cells has not been addressed yet. For example, a conceivable scenario could be that individual cells modified to perform as current collectors by in situ synthesis of FeS nanoparticles (Yu et al., 2020) are embedded in a PEDOT:PSS hydrogel (Zajdel et al., 2018). In another, scenario cells coated with polypyrrole and polydopamine (Wang, Pan, et al., 2021) are embedded in an agarose hydrogel and sprayed (Knoll et al., 2022) onto a carbon paper electrode modified with rGO and silver nanoparticles (Cao et al., 2021). In this way, new ecological niches are colonized by cells with improved adhesion properties, and the overall performance could exceed the individual improvements. In general, it appears that abiotic and biotic strategies are performed and considered independently. So far, mainly wild‐type bacteria have been used for the investigation of optimized technical settings. If both technical and genetic optimization can each achieve higher power and energy yields, it can be hypothesized that a combination would lead to even better performances. For example, as described above, a *S. oneidensis* mutant strain overexpressing *speC* (Edel et al., 2021) colonizing a titanium network to which carbon nanoparticles have been deposited (Pu et al., 2022) would be a conceivable approach for enhancing performance even further. All of the scenarios described are intended to illustrate that MES research must become multidimensional and multidisciplinary so that different perspectives can be considered.

### Studies on scalability and sustainability of improving technologies are necessary

Other important aspects that are unfortunately often not considered are feasibility and sustainability. Various polymer hydrogels or coating techniques are not suitable for large‐scale application as complicated processes are required to obtain the compound, especially in larger quantities. Naturally, lab‐scale studies are rarely about the economics of an application, but a life cycle assessment would be one way of investigating at an early stage which technical modifications might have a realistic chance for scale‐up. These life cycle assessment studies have, e.g., been carried out for wastewater treatment processes (Mathuriya, Hiloidhari, Gware, Singh, & Pant, 2020; Peñacoba‐Antona et al., 2021) and provide information on the economic and ecological viability of the proposed processes. The same applies to the use of carbon or metal‐based materials as the basis for electrodes and to the question of which compounds used for optimization would be economically and ecologically viable. In addition, the sustainability of the aforementioned optimizations should be a considered factor. Both gold and silver nanoparticles, for example, can positively influence the performance of organisms in BES, but few studies consider whether the recovery of these nanoparticles would be possible at all in downstream processing. Without the possibility of resource recovery, there will be hardly any large‐scale industrial application of these nanoparticles.

Another aspect that is rarely addressed is the long‐term stability of the optimized materials and techniques. Mostly, the performance is examined for hours or maybe days. However, for industrial use it would be necessary to investigate whether the performance remains constant over weeks and months. If for instance suspended nanoparticles are added, a short‐term increase is not surprising, but a long‐term increase is questionable since suspended particles are most likely washed out. In this aspect, genetic approaches have a crucial advantage, since genetic modifications of the genome are in most cases very stable and, for example, a deleted gene is irreversibly removed. Further, different technical optimization strategies and their given improvement factors are only partially comparable. As normalization of current and power values are highly dependent on the system used to test the optimizations, no uniform normalization is employed. The improvement factor given for each study is our chosen approach to increase comparability of studies. However, this factor is biased in such a way that it does not cover the native (dis‐)advantage different materials have. As aforementioned, carbon‐based materials could not be as impressively improved as metal‐based electrodes.

## CONCLUDING REMARKS

To sum up, the bacteria–electrode interaction is regarded as one of the major factors limiting the productivity of MESs. Future research and development on electroactive microbial biofilms must combine application‐oriented engineering with fundamental research. To achieve this, studies should be carried out that focus on further improving electricity and energy production as well as on a deeper understanding of EET processes. We propose to combine genetic and engineering techniques, i.e., to use genetically enhanced strains utilizing embedding/coating and electrode modification techniques to improve physical properties.

Finally, MESs as a platform technology has an incredible potential in helping on the way to a sustainable bioeconomy, such as by integrating this technology into wastewater treatment plants or by enabling the production of platform chemicals independently from petroleum‐based processes. Combining optimized biofilm–electrode interaction with improved reactor design and process intensification will allow current conventional techniques to be replaced by MES technology.

## AUTHOR CONTRIBUTIONS

**Edina Marlen Klein:** Conceptualization (equal); visualization (equal); writing – original draft (equal); writing – review and editing (equal). **Melanie Tabea Knoll:** Conceptualization (equal); visualization (equal); writing – original draft (equal); writing – review and editing (equal). **Johannes Gescher:** Conceptualization (equal); funding acquisition (equal); project administration (equal); visualization (equal); writing – review and editing (equal).

## CONFLICT OF INTEREST STATEMENT

The authors declare that a patent application for sprayable, synthetic biofilm hydrogel (patent number 102021105164.9) has been filed. M.T.K. and J.G. are included in the patent and declare competing interest.
